# The LDL Apolipoprotein B-to-LDL Cholesterol Ratio: Association with Cardiovascular Mortality and a Biomarker of Small, Dense LDLs

**DOI:** 10.3390/biomedicines10061302

**Published:** 2022-06-02

**Authors:** Günther Silbernagel, Hubert Scharnagl, Christoph H. Saely, Markus Reinthaler, Martin Rief, Marcus E. Kleber, Barbara Larcher, John Chapman, Juergen R. Schaefer, Heinz Drexel, Winfried März

**Affiliations:** 1 Division of Angiology, Department of Internal Medicine, Medical University of Graz, Auenbruggerplatz 15, 8036 Graz, Austria; guenther.silbernagel@yahoo.com; 2 Clinical Institute of Medical and Chemical Laboratory Diagnostics, Medical University of Graz, Auenbruggerplatz 15, 8036 Graz, Austria; winfried.maerz@synlab.de; 3 Department of Medicine I, Academic Teaching Hospital Feldkirch, Carinagasse 47, 6800 Feldkirch, Austria; christoph.saely@me.com (C.H.S.); barbara.larcher@lkhf.at (B.L.); 4 Vorarlberg Institute for Vascular Investigation and Treatment (VIVIT), Carinagasse 47, 6800 Feldkirch, Austria; vivit@lkhf.at; 5 Private University in the Principality of Liechtenstein, Dorfstr. 24, 9495 Triesen, Liechtenstein; 6 Department of Cardiology, Charité-Universitätsmedizin Berlin (CBF), Hindenburgdamm 30, 12203 Berlin, Germany; markus.reinthaler@charite.de; 7 Institute of Biomaterial Science, Helmholtz-Zentrum Geesthacht, Kantstraße 55, 14513 Teltow, Germany; 8 Division of General Anaesthesiology, Emergency and Intensive Care Medicine, Medical University of Graz, Auenbruggerplatz 5, 8036 Graz, Austria; martin.rief@medunigraz.at; 9 Department of Internal Medicine 5 (Nephrology, Hypertensiology, Endocrinology, Diabetology, Rheumatology), Mannheim Medical Faculty, University of Heidelberg, Theodor-Kutzer-Ufer 1-3, 68167 Mannheim, Germany; marcus.kleber@medma.uni-heidelberg.de; 10 Synlab Human Genetics Laboratory, Synlab AG, Harrlachweg 1, 68163 Mannheim, Germany; 11 Sorbonne University and Pitie-Salpetriere University Hospital, National Institute for Health and Medical Research (INSERM), Boulevard de l’Hopital, 75651 Paris, France; john.chapman@upmc.fr; 12 Center for Undiagnosed and Rare Diseases, University Clinic Marburg, Baldinger Str. 1, 35043 Marburg, Germany; juergen.schaefer@mailer.uni-marburg.de; 13 Drexel University College of Medicine, 2900 W Queen Lane, Philadelphia, PA 19129, USA; 14 Department of Internal Medicine, Landeskrankenhaus Bregenz, Carl-Pedenz-Straße 2, 6900 Bregenz, Austria; 15 Synlab Academy, Synlab Holding Germany GmbH, P5, 7, 68167 Mannheim, Germany

**Keywords:** apolipoprotein B, cardiovascular mortality, cholesterol, small, dense LDLs

## Abstract

Background and Objective: Small, dense low-density lipoproteins (LDLs) are considered more atherogenic than normal size LDLs. However, the measurement of small, dense LDLs requires sophisticated laboratory methods, such as ultracentrifugation, gradient gel electrophoresis, or nuclear magnetic resonance. We aimed to analyze whether the LDL apolipoprotein B (LDLapoB)-to-LDL cholesterol (LDLC) ratio is associated with cardiovascular mortality and whether this ratio represents a biomarker for small, dense LDLs. Methods: LDLC and LDLapoB were measured (beta-quantification) and calculated (according to Friedewald and Baca, respectively) for 3291 participants of the LURIC Study, with a median (inter-quartile range) follow-up for cardiovascular mortality of 9.9 (8.7–10.7) years. An independent replication cohort included 1660 participants. Associations of the LDLapoB/LDLC ratio with LDL subclass particle concentrations (ultracentrifugation) were tested for 282 participants. Results: In the LURIC Study, the mean (standard deviation) LDLC and LDLapoB concentrations were 117 (34) and 85 (22) mg/dL, respectively; 621 cardiovascular deaths occurred. Elevated LDLapoB/LDLC (calculated and measured) ratios were significantly and independently associated with increased cardiovascular mortality in the entire cohort (fourth vs. first quartile: hazard ratio (95% confidence interval) = 2.07 (1.53–2.79)) and in statin-naïve patients. The association between calculated LDLapoB/LDLC ratio and cardiovascular mortality was replicated in an independent cohort. High LDLapoB/LDLC ratios were associated with higher LDL5 and LDL6 concentrations (both *p* < 0.001), but not with concentrations of larger LDLs. Conclusions: Elevated measured and calculated LDLapoB/LDLC ratios are associated with increased cardiovascular mortality. Use of LDLapoB/LDLC ratios allows estimation of the atherogenic risk conferred by small, dense LDLs.

## 1. Introduction

The intimal penetration and retention of low-density lipoproteins (LDLs) represents a key step in the pathophysiological process of atherogenesis [1]. Low-density lipoprotein-cholesterol (LDLC) is considered to reflect the magnitude of this process [1]. Consistently, high LDLC is associated with increased cardiovascular risk [2]; on the other hand, therapeutic lowering of LDLC reduces this risk [3]. Therefore, LDLC is frequently employed in guidelines for the management of dyslipidaemias, as typified and endorsed by the European Society of Cardiology/European Atherosclerosis Society [4], the American College of Cardiology, and the American Heart Association [5]. Not only the circulating concentration of LDLs, but also the chemical composition and the subclass particle profile has an impact on LDL atherogenicity [1]. In this respect, elevated concentrations of small, dense LDLs are associated with increased cardiovascular risk [1,6,7,8]. Quantifying small, dense LDLs, however, requires sophisticated and laborious laboratory methods, potentially involving their ultracentrifugal isolation, or indirect determination by gradient gel electrophoresis or nuclear magnetic resonance spectroscopy [9].

Apolipoprotein B (ApoB) is a direct measure of the particle concentration of all atherogenic lipoproteins, including LDLs, Lp(a), and triglyceride-rich lipoproteins and their remnants, but does not differentiate between LDLs and very low-density lipoproteins (VLDL) [9]. Baca and Warnick have proposed a method for the estimation of LDL apolipoprotein B (LDLapoB) from total apoB [10]. Although LDLapoB represents the concentration of atherogenic LDL particles in all subclasses, it does not differentiate between small, dense LDL particles and larger and lighter subclasses. Our working hypothesis was that an elevated ratio of LDLapoB to LDLC (LDLapoB/LDLC) would be associated with increased cardiovascular risk and that this ratio may be a measure of the concentration of small, dense LDLs.

Therefore, the aim of the present work was to explore the relationship between LDLapoB/LDLC ratios, both measured (LDLapoB/LDLC_meas_) and calculated (LDLapoB/LDLC_calc_), and cardiovascular mortality in participants of the prospective Ludwigshafen Risk and Cardiovascular Health (LURIC) Study (11–13) and to replicate findings in an independent cohort (LDLapoB/LDLC_calc_). Subsequently, it was intended that the question of whether the LDLapoB/LDLC ratio might represent a biomarker for small, dense LDL particles would be examined.

## 2. Material and Methods

### 2.1. Design, Participants, and Clinical Characterization of the LURIC Cohort

A total of 3316 patients who were referred for coronary angiography to the Ludwigshafen Heart Center in Southwest Germany, were recruited between July 1997 and January 2000 [11,12,13]. Inclusion criteria were: German ancestry, clinical stability except for acute coronary syndromes, and the availability of a coronary angiogram. Indications for angiography in individuals with clinically stable disease were chest pain and/or noninvasive test results suggestive of myocardial ischemia. Individuals suffering from any acute illness other than acute coronary syndromes, chronic non-cardiac diseases, or malignancy within the five past years, and those unable to understand the purpose of the study were excluded [11,12,13]. Subjects with missing data were also ruled out, resulting in a subgroup of 3291 participants for the present analysis. Coronary artery disease was diagnosed by angiography. Acute myocardial infarction was defined as a myocardial infarction that had occurred within four weeks prior to enrolment. ST-elevation myocardial infarction was diagnosed if typical electrocardiogram changes were present along with prolonged chest pain refractory to sublingual nitrates and/or enzyme or troponin T elevations. Non-ST-elevation myocardial infarction was diagnosed, if symptoms and troponin T criteria, but not the electrocardiogram criteria for ST-elevation myocardial infarction, were met. The functional capacity of patients with cardiac disease, especially heart failure, was estimated according to a classification developed by the New York Heart Association. Left ventricular function was estimated using echocardiography [11]. Diabetes mellitus was diagnosed according to the current guidelines of the American Diabetes Association [14]. Hypertension was diagnosed when the systolic and/or diastolic blood pressure exceeded 140 and/or 90 mmHg or if there was a history of hypertension, evident from treatment with antihypertensive drugs [11].

### 2.2. Follow-Up of the LURIC Cohort

Information on the vital status was obtained from local population registries. Cardiovascular mortality was defined as death due to fatal myocardial infarction, sudden cardiac death, death after cardiovascular intervention, stroke, or other causes of death due to cardiovascular diseases. An assessment of death certificates was carried out by two experienced clinicians. In a few cases of disagreement or uncertainty concerning the coding of a specific cause of death, classification was made by a principal investigator of the LURIC study [12,13]. The median (interquartile range) duration of follow-up was 9.9 (8.7–10.7) years (mean and standard deviation: 8.8 (3.0)).

### 2.3. Replication Cohort

The replication cohort included 1660 Caucasian patients, of whom 20 were lost during follow-up. They were referred to elective coronary angiography for the evaluation of established or suspected stable coronary artery disease at the academic teaching hospital Feldkirch, a tertiary care centre in Western Austria between 1999 and 2008 [15]. Coronary artery disease was diagnosed by coronary angiography. Diabetes mellitus was diagnosed according to the current guidelines of the American Diabetes Association [14]. Hypertension was defined according to the 2013 European Atherosclerosis Society/European Society of Hypertension guidelines [16]. There was a follow-up for cardiovascular mortality with a median (interquartile range) duration of 10.9 (7.7–12.1) years. Time and causes of death were obtained from a national survey (Statistik Austria, Vienna, Austria) or from hospital records.

### 2.4. Cohorts for Evaluation of LDL Subfractions

We report on data from 282 subjects from 3 studies [17,18,19]. The first study included samples from 106 persons with increased risk for type 2 diabetes or newly diagnosed type 2 diabetes not yet receiving oral antidiabetics, insulin, or lipid-lowering agents. The samples were taken as part of the baseline screening examination for a randomized controlled statin trial [17]. The second study included samples from 77 patients with coronary heart disease or high cardiovascular risk with well-controlled LDLC concentrations. The samples were taken as part of the baseline examination of a randomized controlled trial of a combination treatment of statin and ezetimibe versus statin treatment alone [18]. The third study included samples from 99 patients with type 2 diabetes. The samples were taken as part of the baseline examination of a dietary intervention study comparing a low-carbohydrate diet with a standard diet [19].

### 2.5. Laboratory Analyses

#### 2.5.1. Standard Measurements

All analyses were performed using fasting blood samples. In the LURIC cohort, lipoproteins were separated using a combined ultracentrifugation–precipitation method (β-quantification). The VLDL fraction (*d* < 1.006 g/mL) was recovered by ultracentrifugation (18 h, 10 °C, 30,000 rpm). ApoB-containing lipoproteins in the resulting bottom fraction were precipitated using phosphotungstic acid, with the HDL particles remaining in solution. LDLapob and LDLC were then derived from the substraction of apoB and cholesterol after precipitation from their respective concentrations before precipitation. LDLC was calculated according to Friedewald et al. (LDLC = total cholesterol − HDL cholesterol − triglycerides/5) [20], and LDLapoB was calculated according to Baca and Warnick (LDLapoB = apoB − 10 − triglycerides/32; validated for subjects with triglycerides < 400 mg/dL) [10]. Cholesterol and triglycerides were measured with enzymatic reagents from WAKO (Neuss, Germany) using a WAKO 30 R analyzer. ApoB was measured by turbidimetry (Rolf-Greiner Biochemica, Flacht, Germany). LDL particle diameter was calculated as previously described [21]. C-reactive protein was measured was measured by immunonephelometry on a Behring Nephelometer II (N High Sensitivity CRP, Dade Behring, Marburg, Germany). Glucose was measured with an enzymatic assay on a Hitachi 717 analyzer.

In the replication cohort, serum levels of triglycerides, total cholesterol, LDLC, and HDL cholesterol were measured using enzymatic hydrolysis and precipitation techniques (triglycerides GPO-PAP, CHOD/PAP, QuantolipLDL, QuantolipHDL; Roche, Basel, Switzerland) with a Hitachi-Analyzer 717 or 911. Serum apolipoprotein B was determined on a Cobas Integra 800^®^ (Roche).

#### 2.5.2. Equilibrium Density Gradient Ultracentrifugation of LDL Subfractions

Lipoproteins were isolated by sequential preparative ultracentrifugation using the following densities: *d* < 1.006 kg/L for VLDL, 1.006 < *d* < 1.019 kg/L for intermediate density lipoproteins, 1.019 < *d* < 1.063 kg/L for LDL, and 1.063 < *d* < 1.21 kg/L for HDL. The subfractions of LDLs were separated according to Baumstark et al. into 6 density classes by equilibrium density gradient centrifugation. The density ranges of LDL subfractions were: LDL1, <1.031 kg/L; LDL2, 1.031–1.034 kg/L; LDL3, 1.034–1.037 kg/L; LDL4, 1.037–1.040 kg/L; LDL5, 1.040–1.044 kg/L; LDL6, >1.044 kg/L [22]. The LDL5 and LDL6 fractions were considered small, dense LDLs.

### 2.6. Statistical Analysis

Quartiles of the LDLapoB/LDLC_meas_ and LDLapoB/LDLC_calc_ ratios were formed. Baseline characteristics of the LURIC cohort are presented according to quartiles of LDLapoB/LDLC_meas_ and LDLapoB/LDLC_calc_, in the entire cohort, in statin-naïve patients, and in patients on statins. The χ^2^-test and Analysis of Variance were used to compare the distributions of variables across the quartiles of LDLapoB/LDLC_meas_ and LDLapoB/LDLC_calc_ ratios. Triglycerides and C-reactive protein were transformed logarithmically before being used in parametric statistical models. Pearson correlation was calculated for the relationship between LDLapoB/LDLC_meas_ and LDLapoB/LDLC_calc_ and this relationship was also illustrated using a box and scatter plot. The association of LDLapoB/LDLC_meas_ quartiles with the risk of cardiovascular death in the entire LURIC cohort was examined with Kaplan–Meier curves and with the log-rank test. Cox regression was used to examine the association of LDLapoB/LDLC_meas_ and LDLapoB/LDLC_calc_ quartiles with time to endpoints in the entire LURIC cohort, in statin-naïve patients and in patients on statins using 2 predefined models of adjustment: Model 1 included the covariates sex, age, statin use, and the interaction term between statin use and the LDLapoB/LDLC_meas_ and LDLapoB/LDLC_calc_ quartiles. Model 2 additionally included body mass index, hypertension, diabetes mellitus, smoking, HDL cholesterol, and triglycerides. The analyses in the replication cohort were pre-specified and in accordance with the LURIC study. ANOVA was used to compare the distributions of apoB in LDL subclasses across the quartiles of LDLapoB/LDLC_meas_ and LDLapoB/LDLC_calc_ in the combined cohort with LDL density gradient ultracentrifugation. All statistical tests were 2-sided and *p*-values < 0.05 were considered significant. The SPSS 26 statistical package (SPSS Inc., Chicago, IL, USA) was used.

### 2.7. Ethical Aspects

The LURIC study was approved by the ethics committee of the Physicians Chamber of Rheinland-Pfalz. The replication cohort and the 3 studies, in which LDL subfractions were measured using density gradient ultracentrifugation, were also approved by local ethics committees. All studies were performed in accordance with the declaration of Helsinki, and all participants gave written informed consent [11,12,13,16,17,18,19].

## 3. Results

### 3.1. Baseline Characteristics of the LURIC and Replication Cohorts

The mean (standard deviation) age of the 3291 LURIC participants (2294 males, 997 females) was 62.6 (10.6) years. Participants displayed a mean body mass index of 27.5 (4.1) kg/m^2^ and total and LDLC levels of 192 (39) and 117 (34) mg/dL, respectively. Mean total and LDLapoB values were 104 (25) and 85 (22) mg/dL, respectively. The mean (standard deviation) ratio of LDLapoB/LDLC_meas_ was 0.74 (0.09). Higher LDLapoB/LDLC_meas_ quartiles were positively associated with male sex, body mass index, hypertension, diabetes mellitus, and smoking. Age was inversely related to the quartiles of LDLapoB/LDLC_meas_. Total cholesterol, LDL-cholesterol, HDL-cholesterol, and LDL diameter were inversely related to quartiles of LDLapoB/LDLC_meas._ Total apoB and LDL triglyceride levels were positively related to the quartiles of LDLapoB/LDLC_meas_. Coronary artery disease, impaired left ventricular function, and peripheral artery disease were more prevalent in patients with elevated LDLapoB/LDLC_meas_ ratios. Statin use was more frequent in patients with high LDLapoB/LDLC_meas_ (Table 1). The baseline characteristics according to the quartiles of the LDLapoB/LDLC_meas_ ratio stratified for statin treatment are shown in the Supplementary Materials (Supplementary Tables S1 and S2). Consistent results were obtained for LDLapoB/LDLC_calc_ ratios (Supplementary Tables S3–S5). The baseline characteristics of the replication cohort were similar to the LURIC study (Supplementary Table S6).

### 3.2. Comparison of Measured and Calculated LDLC and LDLapoB

**Figure 1:**
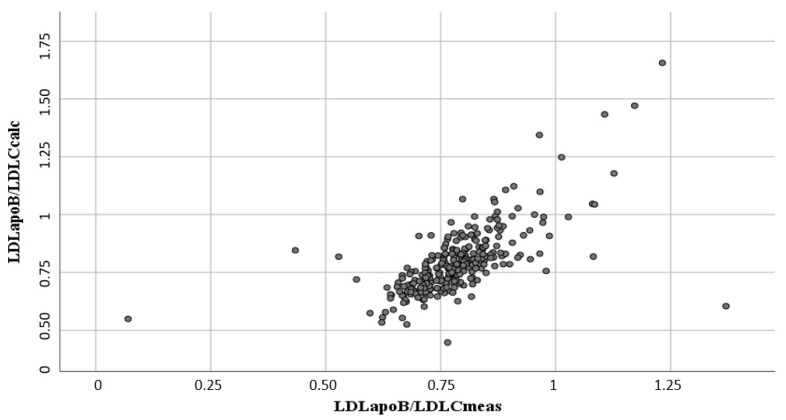
Association between LDLapoB/LDLC_meas_ and LDLapoB/LDLC_calc_ in the LURIC cohort. Legend: for each patient one grey circle.

The mean measured LDLC and LDLapoB concentrations for patients with triglyceride levels of <400 mg/dL were 118 (34) and 85 (22) mg/dL, respectively. In the same subgroup, the LDLC and LDLapoB concentrations, calculated according to Friedewald et al. [20] and Baca et al. [10], respectively, were 121 (34) and 89 (24) mg/dL, respectively. The correlations between the measured and calculated LDLC and LDLapoB concentrations were r = 0.90, *p* < 0.001 and r = 0.91, *p* < 0.001, respectively. The correlation between LDLapoB/LDLC_meas_ and LDLapoB/LDLC_calc_ (LDLapoB/LDLC_calc_ = 0.132 + 0.842 × LDLapoB/LDLC_meas_) was r = 0.73, *p* < 0.001 (Figure 1).

### 3.3. LDLapoB/LDLC_meas_ and LDLapoB/LDLC_calc_ Ratio and Cardiovascular Mortality

**Figure 2:**
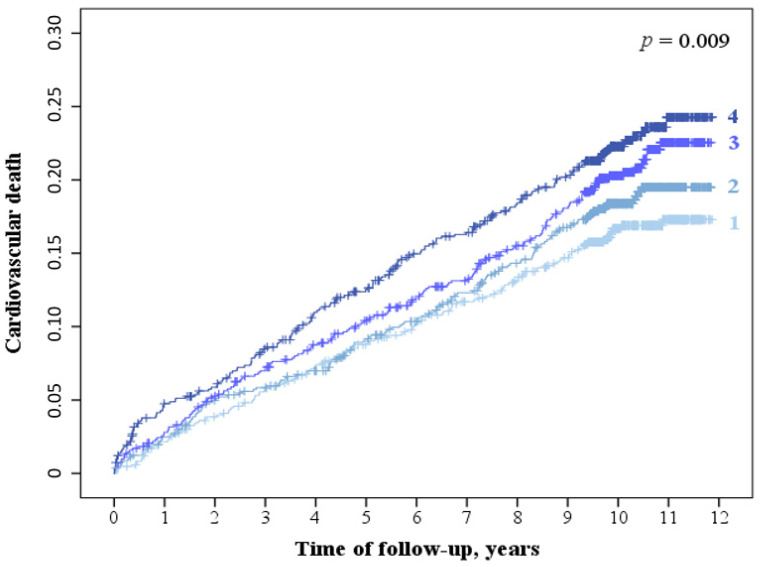
Kaplan-Meier curves for cardiovascular death according to LDLapoB/LDLC_meas_ quartiles in the entire LURIC cohort. Legend: 1-4: LDLapoB/LDLC_meas_ quartiles; p calculated with log-rank test.

A total of 621 cardiovascular deaths occurred during the follow-up. The quartiles of LDLapoB/LDLC_meas_ were positively related to cardiovascular mortality (Figure 2) adjusted for sex, age, statin treatment, and interaction between statin treatment and LDLapoB/LDLC_meas_ quartiles (Table 2). The association of the LDLapoB/LDLC_meas_ quartiles with cardiovascular mortality remained significant after multivariate adjustment (Table 2). There was significant interaction between LDLapoB/LDLC_meas_ quartiles and statin use in predicting cardiovascular mortality (*p* = 0.030). Stratified analyses revealed that quartiles of LDLapoB/LDLC_meas_ ratios were strongly and positively associated with cardiovascular mortality in statin-naïve patients adjusted for sex and age and after multivariate adjustment (Table 2). In contrast, quartiles of LDLapoB/LDLC_meas_ ratios were not associated with cardiovascular mortality in patients receiving statin treatment (Table 2). Consistent results were obtained for LDLapoB/LDLC_calc_ (Table 3). In agreement with the LURIC study, high LDLapoB/LDLC_calc_ quartiles were associated with increased cardiovascular mortality in the replication cohort (Supplementary Table S7). The sample size and the number of events were low for subgroup analyses.

### 3.4. LDLapoB/LDLC_meas_, LDLapoB/LDLC_calc_ and LDL Subclass Particle Concentrations in the LDL Subfraction Cohort

The mean (standard deviation) LDLC and LDLapoB concentrations were 110 (36) and 86 (35) mg/dL, respectively. Higher quartiles of LDLapoB/LDLC_meas_ were associated with increased apoB concentration in the dense LDL fraction (LDL5 and LDL6) but were not associated with apoB concentration in the larger LDL fractions (Table 4). Consistent results were obtained for ratios of LDLapoB/LDLC_calc_ in the subgroup with triglyceride levels <400 mg/dL (Table 5).

## 4. Discussion

The main finding in the present study was that elevated values for the ratios of both LDLapoB/LDLC_meas_ and LDLapoB/LDLC_calc_ were associated with increased cardiovascular mortality in LURIC participants and an independent replication cohort. In addition, higher values for LDLapoB/LDLC_meas_ and LDLapoB/LDLC_calc_ were associated with elevated levels of small, dense LDLs and lower mean LDL particle diameters. Therefore, this study has immediate practical implications, as it indicates that LDLapoB/LDLC_calc_ ratios based on the equations proposed by Baca and Warnick [10] and Friedewald et al. [20] may be used to estimate the atherogenic risk associated with small, dense LDLs. Importantly, the ESC/EAS 2019 guidelines for the treatment of dyslipidaemias strongly recommend the use of total apoB as an integral index for the entirety of atherogenic, apoB-containing lipoprotein particles [4]. Hence, information on concentrations of small, dense LDLs can be obtained using the simple LDLapoB/LDLC_calc_ equation at no additional laboratory cost. The required calculations can be readily integrated into any laboratory information system and do not require the use of a sophisticated methodology, such as ultracentrifugation, gradient gel electrophoresis, or nuclear magnetic resonance spectroscopy.

In agreement with the LURIC cohort, several epidemiological studies, such as the Stanford Five-City Project [23] and the Quebec Cardiovascular Study [24], have confirmed positive correlations between levels of small, dense LDL particles and cardiovascular risk. Interestingly, we also observed particularly strong associations between elevated values of LDLapoB/LDLC and increased prevalence of peripheral artery disease. This finding is in agreement with recent findings of the Women’s Health Study [25]. We previously found in the LURIC cohort that not only patients with a mean small LDL particle diameter displayed increased cardiovascular risk; those patients exhibiting predominantly high LDL particle diameters also displayed increased risk [21]. In contrast, there was a continuous increase in cardiovascular risk conferred by increasing values of the LDLapoB/LDLC ratio. Patients with large mean LDL diameter simultaneously display elevated LDL triglyceride concentrations, whereas there was a continuous positive relationship between LDLapoB/LDLC and LDL triglycerides observed. Of relevance, high LDL triglyceride concentrations may potentially reflect the atherogenic effects of low hepatic lipase activity [13].

Small, dense LDL particles are considered highly atherogenic on a per particle basis as a result of several intrinsic features [1]. Firstly, dense LDLs are more likely to penetrate into the sub-intimal space and possess higher affinity for binding to arterial wall proteoglycans, thereby implicating enhanced retention in the sub-intimal space [1]. Small, dense LDLs equally exhibit a prolonged half-life in the circulation due to lower LDL receptor affinity and thus reduced hepatic uptake [26]. Taken together, these features render small, dense LDLs more susceptible to deposition in the arterial wall [27]. Accordingly, this consideration is consistent with the absence of significant associations between LDLapoB/LDLC ratio and cardiovascular mortality for patients under statin treatment. Potentially, increased catabolism of small, dense LDLs [28], induced by higher expression of the hepatic LDL receptor in response to statin medication [29], may have blunted the association of the LDLapoB/LDLC with cardiovascular mortality in these patients. Alternatively, the lack of an association between LDLapoB/LDLC ratio and cardiovascular mortality in statin users may be due to general confounding by lipid-lowering treatment. This has also been observed in other highly recognized epidemiological studies [30].

Small, dense LDLs are also more susceptible to modification by oxidation and glycation of their phospholipid and cholesteryl ester components [1]. Finally, cardiovascular risk associated with small, dense LDLs may be due to an unfavorable lipid composition compared with LDLs of larger size [31].

Another supportive observation was that subjects with high LDLapoB/LDLch ratios showed elevated C-reactive protein levels. Of interest, small, dense LDLs are preferentially enriched with apolipoprotein C-III, which may increase cardiovascular risk [32], not only by inhibition of lipoprotein lipase but also via alternative inflammasome activation [33]. Hence, the well-documented association between small, dense LDLs and inflammation [34] may also be accounted for by the pro-inflammatory effects of apolipoprotein C-III.

This is, to the best of our knowledge, the first prospective study relating ratios of LDLapoB/LDLC_meas_ and LDLapoB/LDLC_calc_ to cardiovascular mortality. The results of the present analyses also extend recent findings from the replication cohort that the LDLC/apoB ratio was inversely related to major cardiovascular events [35]. Moreover, the study includes a comparison of the LDLapoB/LDLC_meas_ and LDLapoB/LDLC_calc_ ratios with direct measurements of LDL particle concentrations. We also performed a precise clinical and biochemical characterization of the study participants. In addition, we have reported on a long-term follow-up with a large number of endpoints yielding high statistical power. Finally, the results were confirmed in an independent replication cohort.

It may be a limitation of the present study that laboratory measurements were performed once at baseline only. Consequently, we were not able to account for possible changes in LDLapoB/LDLC ratios during the follow-up. Moreover, data on non-fatal cardiovascular endpoints were not collected in the LURIC study.

In conclusion, elevated levels of LDL particles relative to their cholesterol content were associated with increased cardiovascular mortality. The LDLapoB/LDLC_calc_ ratio may hold the potential to quantify the atherogenic risk associated with small, dense LDL particles using a simple methodology. Further studies are required, however, to support the use of this ratio in patient cohorts differing in ethnicity, gender, and lipid-lowering pre-treatment.

## Figures and Tables

**Table 1:** Baseline characteristics according to LDLapoB/LDLC_meas_ quartiles in the entire LURIC cohort.

	1st Quartile	2nd Quartile	3rd Quartile	4th Quartile	*p* *
Number	836	810	821	824	-
Male sex	456 (54.5)	570 (70.4)	629 (76.6)	639 (77.5)	<0.001
Age, years	63.3 (10.8)	63.2 (10.6)	62.6 (10.4)	61.4 (10.6)	0.001
Body mass index, kg/m^2^	26.7 (3.9)	27 (3.8)	27.9 (4.2)	28.4 (4.1)	<0.001
Hypertension	572 (68.4)	588 (72.6)	602 (73.3)	629 (76.3)	0.004
Smoking					<0.001
Never	396 (47.4)	287 (35.4)	269 (32.8)	234 (28.4)	
Former	302 (36.1)	367 (45.3)	372 (45.3)	414 (50.2)	
Current	138 (16.5)	156 (19.3)	180 (21.9)	176 (21.4)	
Diabetes mellitus	248 (29.7)	284 (35.1)	365 (44.5)	414 (50.2)	<0.001
Lipids					
Total cholesterol, mg/dL †	207 (39)	192 (34)	187 (37)	182 (42)	<0.001
LDL cholesterol, mg/dL †	135 (37)	121 (29)	114 (30)	95.5 (29)	<0.001
HDL cholesterol, mg/dL †	45.5 (11.5)	41 (10)	36 (9)	32 (8)	<0.001
Triglycerides, mg/dL ‡	119 (45)	140 (55)	170 (64)	264 (184)	<0.001 §
LDL triglycerides, mg/dL ‡	29 (10)	30 (10)	33 (12)	34 (14)	<0.001 §
ApoB, mg/dL	102 (25)	103 (23)	106 (25)	107 (26)	<0.001
LDL apo B, mg/dL	87 (23)	86 (20)	86 (22)	81(23)	<0.001
LDL apolipoprotein B-to-LDL cholesterol ratio	0.65 (0.03)	0.71 (0.01)	0.76 (0.02)	0.86 (0.10)	-
LDL diameter, nm	17.0 (0.4)	16.6 (0.3)	16.5 (0.3)	16.2 (0.4)	<0.001
C-reactive protein, mg/L	6.1 (13.5)	8.1 (15.3)	10.8 (20.9)	11.3 (21.1)	<0.001 §
Coronary artery disease ||					<0.001
No	289 (35)	189 (23.4)	127 (16.2)	128 (16.2)	
Stable angina	372 (45.1)	388 (49.0)	393 (50.1)	378 (47.7)	
ACS	164 (19.9)	215 (27.1)	265 (33.8)	286 (36.1)	
NYHA functional class					0.131
I	432 (51.7)	426 (52.6)	437 (53.2)	415 (50.4)	
II	265 (31.7)	233 (28.8)	229 (27.9)	235 (28.5)	
III	126 (15.1)	125 (15.4)	124 (15.1)	146 (17.7)	
IV	13 (1.6)	26 (3.2)	31 (3.8)	28 (3.4)	
Left ventricular function #					<0.001
Normal	566 (70.8)	502 (64.2)	447 (56.4)	454 (57.2)	
Mildly impaired	99 (12.4)	113 (14.5)	126 (15.9)	122 (15.4)	
Moderately impaired	62 (7.8)	83 (10.6)	103 (13)	102 (12.8)	
Severely impaired	23 (2.9)	36 (4.6)	44 (5.5)	41 (5.2)	
Friesinger score					<0.001
1st quartile	282 (33.7)	176 (21.7)	121 (14.7)	135 (16.4)	
2nd quartile	223 (26.7)	182 (22.5)	168 (20.5)	168 (20.4)	
3rd quartile	202 (24.2)	261 (32.2)	304 (37)	295 (35.8)	
4th quartile	129 (15.4)	191 (23.6)	228 (27.8)	226 (27.4)	
Peripheral vascular disease	49 (5.9)	77 (9.5)	82 (10)	101 (12.3)	<0.001
Cerebrovascular disease	74 (8.9)	62 (7.7)	86 (10.5)	77 (9.3)	0.256
Lipid-lowering drugs					
Statin	231 (27.6)	337 (41.6)	472 (57.5)	503 (61)	<0.001
Non-statin lipid-lowering drugs	17 (2)	21 (2.6)	15 (1.8)	26 (3.2)	0.288

Legend: Values are means ± standard deviations or medians (25th–75th percentiles) in cases of continuous variables and numbers (percentages) in cases of categorical data. * For differences across the four groups calculated with the χ^2^ test and Analysis of Variance for categorical and continuous data, respectively. † To convert to millimoles per liter, multiply by 0.02586. ‡ To convert to millimoles per liter, multiply by 0.01129. § Analysis Of Variance of logarithmically transformed values. || 825/792/785/792. **#** 750/734/720/719.

**Table 2:** Cardiovascular mortality according to LDLapoB/LDLC_meas_ quartiles in the LURIC cohort.

			Model 1 *		Model 2 †	
	N	CD (%)	HR (95% CI)	*p*	HR (95% CI)	*p*
**Entire cohort**						
1st quartile	836	133 (15.9)	1.0 reference	-	1.0 reference	-
2nd quartile	810	145 (17.9)	1.09 (0.81–1.47)	0.575	0.98 (0.72–1.34)	0.914
3rd quartile	821	164 (20.0)	1.62 (1.20–2.18)	0.002	1.32 (0.96–1.82)	0.087
4th quartile	824	179 (21.7)	2.07 (1.53–2.79)	<0.001	1.69 (1.19–2.40)	0.003
**Statin-naïve**						
1st quartile	605	92 (15.2)	1.0 reference	-	1.0 reference	-
2nd quartile	473	79 (16.7)	1.07 (0.80–1.5)	0.631	0.99 (0.73–1.35)	0.949
3rd quartile	349	81 (23.2)	1.6 (1.18–2.16)	0.002	1.38 (0.98–1.93)	0.064
4th quartile	321	84 (26.2)	2.0 (1.51–2.75)	<0.001	1.84 (1.25–2.70)	0.002
**Statins**						
1st quartile	231	41 (17.7)	1.0 reference	-	1.0 reference	-
2nd quartile	337	66 (19.6)	1.09 (0.74–1.62)	0.656	1.01 (0.68–1.50)	0.969
3rd quartile	472	83 (17.6)	1.05 (0.72–1.53)	0.808	0.90 (0.60–1.33)	0.581
4th quartile	503	95 (18.9)	1.25 (0.86–1.81)	0.247	0.97 (0.63–1.49)	0.884

Legend: N, number; CD, cardiovascular death; HR, hazard ratio (calculated with Cox regression); CI, confidence interval. * Adjusted for sex, age, statin use, and interaction between statin use and LDLapoB/LDLC_calc_ quartiles. † Model 1 with additional adjustment for body mass index, hypertension, diabetes, HDL cholesterol, triglycerides, smoking, and use of non-statin lipid-lowering drugs.

**Table 3:** Cardiovascular mortality according to LDLapoB/LDLC_calc_ quartiles in the LURIC cohort.

			Model 1 *		Model 2 †	
	N	CD (%)	HR (95% CI)	*p*	HR (95% CI)	*p*
**Entire cohort**						
1st quartile	792	118 (14.9)	1.0 reference	-	1.0 reference	-
2nd quartile	793	148 (18.7)	1.48 (1.09–2.00)	0.012	1.41 (1.03–1.92)	0.032
3rd quartile	795	167 (21.0)	1.71 (1.25–2.34)	0.001	1.47 (1.04–2.06)	0.028
4th quartile	793	162 (20.4)	1.83 (1.33–2.52)	<0.001	1.66 (1.12–2.44)	0.011
**Statin-naïve**						
1st quartile	570	80 (14.0)	1.0 reference	-	1.0 reference	-
2nd quartile	462	89 (19.3)	1.46 (1.08–1.98)	0.014	1.48 (1.08–2.04)	0.015
3rd quartile	348	78 (22.4)	1.69 (1.24–2.32)	0.001	1.61 (1.12–2.31)	0.011
4th quartile	311	72 (23.2)	1.81 (1.31–2.49)	<0.001	1.99 (1.29–3.07)	0.002
**Statins**						
1st quartile	222	38 (17.1)	1.0 reference	-	1.0 reference	-
2nd quartile	331	59 (17.8)	1.15 (0.77–1.74)	0.493	1.08 (0.72–1.65)	0.703
3rd quartile	447	89 (19.9)	1.34 (0.91–1.97)	0.133	1.14 (0.76–1.71)	0.531
4th quartile	482	90 (18.7)	1.37 (0.93–2.00)	0.110	1.02 (0.64–1.63)	0.927

Legend: N, number; CD, cardiovascular death; HR, hazard ratio (calculated with Cox regression); CI, confidence interval. * Adjusted for sex, age, statin use, and interaction between statin use and LDLapoB/LDLC_calc_ quartiles. † Model 1 with additional adjustment for body mass index, hypertension, diabetes, HDL cholesterol, triglycerides, smoking, and use of non-statin lipid-lowering drugs.

**Table 4:** LDLapoB in LDL subclasses according to LDLapoB/LDLC_meas_ quartiles.

	1st Quartile	2nd Quartile	3rd Quartile	4th Quartile	*p* *
N	70	69	73	70	-
LDL1apoB, mg/dL	11.1 (5.9)	10.3 (6.0)	9.4 (5.2)	10.6 (9.7)	0.544
LDL2apoB, mg/dL	8.9 (4.7)	7.6 (4.1)	7.0 (3.9)	7.2 (4.8)	0.053
LDL3apoB, mg/dL	12.0 (5.9)	11.0 (5.6)	10.0 (5.1)	9.9 (5.6)	0.095
LDL4apoB, mg/dL	14.2 (6.7)	16.0 (7.0)	14.9 (6.6)	9.9 (5.6)	0.549
LDL5apoB, mg/dL	12.6 (6.8)	17.3 (8.2)	17.7 (9.4)	19.6 (11.4)	<0.001
LDL6apoB, mg/dL	11.2 (5.0)	15.9 (8.4)	17.8 (8.8)	22.1 (14.0)	<0.001

Legend: Values are means (standard deviations); lipoproteins were isolated by ultracentrifugation. The subfractions of LDLs were separated into six density classes by equilibrium density gradient centrifugation: LDL1, <1.031 kg/L; LDL2, 1.031–1.034 kg/L; LDL3, 1.034–1.037 kg/L; LDL4, 1.037–1.040 kg/L; LDL5, 1.040–1.044 kg/L; LDL6, >1.044 kg/L. LDL5 and LDL6 were considered small, dense LDLs. * For trends calculated with Analysis of Variance.

**Table 5:** LDLapoB in LDL subclasses according to LDLapoB/LDLC_calc_ quartiles.

	1st Quartile	2nd Quartile	3rd Quartile	4th Quartile	*p* *
N	66	67	67	67	-
LDL1apoB, mg/dL	10.3 (6.9)	11.1 (6.2)	9.8 (5.2)	9.4 (5.7)	0.389
LDL2apoB, mg/dL	7.9 (4.7)	8.6 (4.3)	7.3 (3.9)	6.0 (4.2)	0.122
LDL3apoB, mg/dL	10.3 (5.4)	12.5 (5.9)	10.5 (5.3)	10.1 (5.4)	0.043
LDL4apoB, mg/dL	12.6 (6.5)	16.4 (6.7)	16.1 (7.5)	15.2 (7.1)	0.007
LDL5apoB, mg/dL	12.3 (9.6)	17.2 (8.5)	18.4 (9.1)	18.9 (9.1)	<0.001
LDL6apoB, mg/dL	11.2 (7.4)	15.5 (9.0)	18.4 (9.0)	20.2 (11.3)	<0.001

Legend: Values are means (standard deviations). Lipoproteins were isolated by ultracentrifugation. The subfractions of LDLs were separated according into six density classes by equilibrium density gradient centrifugation: LDL1, <1.031 kg/L; LDL2, 1.031–1.034 kg/L; LDL3, 1.034–1.037 kg/L; LDL4, 1.037–1.040 kg/L; LDL5, 1.040–1.044 kg/L; LDL6, >1.044 kg/L; LDL5 and LDL6 were considered, dense LDLs. * For trends calculated with Analysis of Variance.

## Data Availability

Data may be made available upon request.

## Supplementary Materials

The following supporting information can be downloaded at: https://www.mdpi.com/article/10.3390/biomedicines10061302/s1.

## Abbreviations

LDL	Low-density lipoprotein
VLDL	Very low-density lipoprotein
HDL	High-density lipoprotein
apoB	Apolipoprotein B
LDLC	LDL cholesterol
LDLapoB	LDL apolipoprotein B
LURIC	Ludwigshafen Risk and Cardiovascular Health
