# Supplementary material for: The LDL Apolipoprotein B-to-LDL Cholesterol Ratio: Association with Cardiovascular Mortality and a Biomarker of Small, Dense LDLs
Source: Biomedicines. 2022 Jun 2;10(6):1302. doi: 10.3390/biomedicines10061302 (PMC9220033; doi:10.3390/biomedicines10061302)
Supplement: Supplementary file 1 [file biomedicines-10-01302-s001.zip › biomedicines-1740941-supplementary.pdf]

The LDL-Apolipoprotein B to LDL-Cholesterol Ratio: Association with Cardiovascular Mortality and a Biomarker of Small Dense LDL

Silbernagel et al.: Calculated Small Dense LDL and Cardiovascular Risk

Günther Silbernagel, MD[a], Hubert Scharnagl, PhD[b]*, Christoph Saely, MD [c, d], Markus Reinthaler MD[e, f], Martin Rief, MD[g], Marcus E. Kleber, PhD[h, i], Barbara Larcher, MD[c, d], M. John Chapman, PhD/Dsc[j], Juergen R. Schaefer, MD[k], Heinz Drexel, MD[d, l, m, n]
Winfried März, MD[b, h, o]

Medical University of Graz, Austria

[a]Division of Angiology, Department of Internal Medicine, Medical University of Graz; Auenbruggerplatz 15, 8036 Graz, Austria
[b] Clinical Institute of Medical and Chemical Laboratory Diagnostics, Medical University of Graz; Auenbruggerplatz 15, 8036 Graz, Austria
[c] Department of Medicine I, Academic Teaching Hospital Feldkirch, Carinagasse 47, Feldkirch, 6800, Austria
[d] Vorarlberg Institute for Vascular Investigation and Treatment (VIVIT), Carinagasse 47, Feldkirch, 6800, Austria.
[e] Department of Cardiology, Charité-Universitätsmedizin Berlin (CBF), Hindenburgdamm 30, 12203 Berlin, Germany
[f] Institute of Biomaterial Science, Helmholtz-Zentrum Geesthacht, Kantstraße 55, 14513 Teltow, Germany
[g] Division of General Anaesthesiology, Emergency and Intensive Care Medicine, Medical University of Graz, Auenbruggerplatz 5, 8036, Graz, Austria
[h] Department of Internal Medicine 5 (Nephrology, Hypertensiology, Endocrinology, Diabetology, Rheumatology), Mannheim Medical Faculty, University of Heidelberg, Theodor-Kutzer-Ufer 1-3, 68167 Mannheim, Germany
[i] Synlab Human Genetics Laboratory, Synlab AG, Harrlachweg 1, 68163 Mannheim
[j] Sorbonne University and Pitie-Salpetriere University Hospital, and National Institute for Health and Medical Research (INSERM), e83, Boulevard de l'Hopital, 75651 Paris, France.
[k] Center for Undiagnosed and Rare Diseases, University Clinic Marburg, Baldinger Str. 1, D-35043, Marburg, Germany
[l] Private University of the Principality of Liechtenstein, Dorfstr. 24, Triesen, 9495, Liechtenstein.
[m] Drexel University College of Medicine, 2900 W Queen Lane, Philadelphia, PA, 19129, USA
[n] Department of Internal Medicine, Landeskrankenhaus Bregenz, Carl-Pedenz-Straße 2, 6900, Bregenz, Austria[o] Synlab Academy, Synlab Holding Germany GmbH, P5, 7, 68167, Mannheim, Germany

Supplementary Materials

Supplementary Table S1

Title: Baseline characteristics according to LDLapoB/$LDLC_{meas}$ quartiles in statin-naïve LURIC patients

	1st quartile	2nd quartile	3rd quartile	4th quartile	*p**
**Number**	605	473	349	321	-
**Male sex**	327 (54)	330 (69.8)	262 (75.1)	250 (77.9)	<0.001
**Age**, years	62.8 (11.1)	62.6 (11.5)	62.7 (10.8)	61.5 (10.8)	0.372
**Body mass index**, kg/m^2	26.7 (3.9)	27 (4)	28.1 (4.4)	28.7 (4.2)	<0.001
**Hypertension**	412 (68.1)	350 (74.0)	260 (74.5)	253 (78.8)	0.004
**Smoking**					<0.001
Never	299 (49.4)	180 (38.1)	124 (35.5)	100 (31.2)	
Former	210 (34.7)	202 (42.7)	162 (46.4)	151 (47.0)	
Current	96 (15.9)	91 (19.2)	63 (18.1)	70 (21.8)	
**ADA 2010**	175 (28.9)	162 (34.2)	170 (48.7)	161 (50.2)	<0.001
**Lipids §**					
Total cholesterol, mg/dl†	210 (37)	196 (32)	197 (36)	194 (40)	<0.001
LDL cholesterol, mg/dl†	137 (34)	124 (27)	122 (27)	102 (29)	<0.001
HDL cholesterol, mg/dl†	46 (12)	41 (10)	36 (9)	32.6 (8.5)	<0.001
Triglycerides, mg/dl ‡	118 (44)	140 (52)	182 (68)	279 (170)	<0.001§
LDL triglycerides, mg/dl‡	29 (10)	30 (10)	34 (12)	36 (15)	<0.001§
Total apolipoprotein B, mg/dl	103 (23)	105 (21)	113 (24)	114 (25)	<0.001
LDL apolipoprotein B, mg/dl	88 (22)	88 (19)	92 (21)	86 (22)	0.004
LDL apolipoprotein B to LDL cholesterol ratio	0.65 (0.03)	0.71 (0.01)	0.75 (0.02)	0.86 (0.13)	-
LDL diameter, nm	16.9 (0.4)	16.6 (0.3)	16.5 (0.3)	16.2 (0.5)	<0.001
**C-reactive protein**, mg/l	5.4 (11.4)	8.0 (16.1)	9.7 (16.9)	9.4 (17.3)	<0.001§
**Coronary artery disease** ‖					<0.001
No	251 (41.8)	168 (36)	98 (28.8)	94 (29.9)	
Stable angina	259 (43.2)	204 (43.7)	157 (46.2)	155 (49.4)	
Acute coronary syndrome	90 (15)	95 (20.3)	85 (25)	65 (20.7)	
**NYHA functional class**					0.393
I	311 (51.4)	239 (50.5)	168 (48.1)	154 (48)	
II	194 (32.1)	140 (29.6)	113 (32.4)	97 (30.2)	
III	88 (14.5)	79 (16.7)	53 (15.2)	56 (17.4)	
IV	12 (2.0)	15 (3.2)	15 (4.3)	14 (4.4)	
**Left ventricular function #**					<0.001
Normal	429 (74.4)	304 (66.8)	190 (58.1)	183 (60.4)	
Midly impaired	65 (11.3)	63 (13.8)	45 (13.8)	44 (14.5)	
Moderately impaired	40 (6.9)	44 (9.7)	41 (12.5)	38 (12.5)	
Severely impaired	15 (2.6)	22 (4.8)	26 (8)	20 (6.6)	
**Friesinger score**					<0.001
1st quartile	242 (40)	150 (31.7)	96 (27.5)	100 (31.2)	
2nd quartile	176 (29.1)	132 (27.9)	95 (27.2)	74 (23.1)	
3rd quartile	125 (20.7)	115 (24.3)	90 (25.8)	84 (26.2)	
4th quartile	62 (10.2)	76 (16.1)	68 (19.5)	63 (19.6)	
**Peripheral vascular disease**	27 (4.5)	37 (7.8)	28 (8)	40 (12.5)	<0.001
**Cerebrovascular disease**	51 (8.4)	37 (7.8)	39 (11.2)	27 (8.4)	0.365
**Non-statin lipid lowering drugs**	15 (2.5)	15 (3.2)	12 (3.4)	13 (4)	0.606

Legend: Values are means ± standard deviations or medians (25th-75th percentiles) in cases of continuous variables and numbers (percentages) in cases of categorical data; * for differences across the 4 groups calculated with χ^2 test and ANalysis of VAriance for categorical and continuous data, respectively; † to convert to millimoles per liter, multiply by 0.02586; ‡ to convert to millimoles per liter, multiply by 0.01129; § ANalysis Of VAriance of logarithmically transformed values; ‖ 600/467/340/314; # 549/433/302/285

Supplementary Table S2

Title: Baseline characteristics according to LDLapoB/$LDLC_{meas}$ quartiles in LURIC patients on statins

	1st quartile	2nd quartile	3rd quartile	4th quartile	*p**
**Number**	231	337	472	503	-
**Male sex**	129 (55.8)	240 (71.2)	367 (77.8)	389 (77.3)	<0.001
**Age**, years	64.4 (9.8)	64 (9.1)	62.7 (10.1)	61.3 (10.4)	<0.001
**Body mass index**, kg/m^2	26.8 (4.1)	27.2 (3.5)	27.7 (4)	28.2 (4.1)	<0.001
**Hypertension**	160 (69.3)	238 (70.6)	342 (72.5)	376 (74.8)	0.384
**Smoking**					0.001
Never	97 (42)	107 (31.8)	145 (30.7)	134 (26.6)	
Former smoker	92 (39.8)	165 (49)	210 (44.5)	263 (52.3)	
Current smoker	42 (18.2)	65 (19.3)	117 (24.8)	106 (21.1)	
**Diabetes mellitus**	73 (31.6)	122 (36.2)	195 (41.3)	253 (50.3)	<0.001
**Lipids**					
Total cholesterol, mg/dl†	201 (44)	187 (35)	180 (35)	175 (41)	<0.001
LDL cholesterol, mg/dl†	130 (42)	117 (32)	109 (30)	91 (28)	<0.001
HDL cholesterol, mg/dl†	44 (11)	40 (9)	36.7 (9.5)	32 (7)	<0.001
Triglycerides, mg/dl ‡	122 (48)	139 (60)	161 (60)	255 (193)	<0.001§
LDL triglycerides, mg/dl‡	31(11)	30 (10)	32 (11)	32 (13)	0.257§
Total apolipoprotein B, mg/dl	99.5 (27.6)	99.7 (24)	101 (24)	102 (26)	0.384
LDL apolipoprotein B, mg/dl	84 (27)	83 (22)	82 (23)	77 (22)	<0.001
LDLapolipoprotein B to LDLcholesterol ratio	0.65 (0.03)	0.71 (0.01)	0.76 (0.02)	0.85 (0.08)	-
LDL diameter, nm	17.0 (0.4)	16.6 (0.3)	16.5 (0.3)	16.2 (0.3)	<0.001
**C-reactive protein**, mg/l	7.7 (17.7)	8.2 (14.3)	11.6 (23.3)	12.5 (23.7)	<0.001§
**Coronary artery disease ||**					<0.001
No	38 (16.9)	21 (6.5)	29 (6.5)	34 (7.1)	
Stable Angina	113 (50.2)	184 (56.6)	236 (53.0)	223 (46.7)	
ACS	74 (32.9)	120 (36.9)	180 (40.4)	221 (46.2)	
**NYHA functional class**					0.220
I	121 (52.4)	187 (55.5)	269 (57)	261 (51.9)	
II	71 (30.7)	93 (27.6)	116 (24.6)	138 (27.4)	
III	38 (16.5)	46 (13.6)	71 (15)	90 (17.9)	
IV	1 (0.4)	11 (3.3)	16 (3.4)	14 (2.8)	
**Left ventricular function #**					0.884
Normal	137 (61.4)	198 (60.6)	257 (55.2)	271 (55.2)	
Midly impaired	34 (15.2)	50 (15.3)	81 (17.4)	78 (15.9)	
Moderately impaired	22 (9.9)	39 (11.9)	62 (13.3)	64 (13)	
Severely impaired	8 (3.6)	14 (4.3)	18 (3.9)	21 (4.3)	
**Friesinger score**					<0.001
1st quartile	40 (17.3)	26 (7.7)	25 (5.3)	35 (7.0)	
2nd quartile	47 (20.3)	50 (14.8)	73 (15.5)	94 (18.7)	
3rd quartile	77 (33.3)	146 (43.3)	214 (45.3)	211 (41.9)	
4th quartile	67 (29)	115 (34.1)	160 (33.9)	163 (32.4)	
**Peripheral vascular disease**	22 (9.5)	40 (11.9)	54 (11.4)	61 (12.1)	0.770
**Cerebrovascular disease**	23 (10)	25 (7.4)	47 (10)	50 (9.9)	0.576
**Non-statin lipid lowering drugs**	2 (0.9)	6 (1.8)	3 (0.6)	13 (2.6)	0.075

Legend: Values are means ± standard deviations or medians (25th-75th percentiles) in cases of continuous variables and numbers (percentages) in cases of categorical data; * for differences across the 4 groups calculated with χ^2 test and ANalysis of VAriance for categorical and continuous data, respectively; † to convert to millimoles per liter, multiply by 0.02586; ‡ to convert to millimoles per liter, multiply by 0.01129; § ANalysis Of VAriance of logarithmically transformed values; || 225/325/445/478; # 201/301/418/434

Supplementary Table S3

Title: Baseline characteristics according to LDLapoB/LDLC$_{calc}$ quartiles in the entire LURIC cohort

	1st quartile	2nd quartile	3rd quartile	4th quartile	*p**
**Number**	792	793	795	793	-
**Male sex**	337 (42.6)	220 (27.7)	209 (26.3)	202 (25.5)	<0.001
**Age**, years	63.2 (11.1)	62.9 (10.7)	62.7 (10.3)	62.2 (10.3)	0.283
**Body mass index**, kg/m^2	26.4 (3.7)	27.1 (3.9)	27.7 (4.1)	28.6 (4.3)	<0.001
**Hypertension**	538 (67.9)	555 (70.0)	584 (73.5)	616 (77.7)	<0.001
**Smoking**					<0.001
Never	373 (47.1)	293 (36.9)	262 (33.0)	228 (28.8)	
Former	295 (37.2)	352 (44.4)	369 (46.4)	382 (48.2)	
Current	124 (15.7)	148 (18.7)	164 (20.6)	183 (23.1)	
**ADA 2010**	209 (26.4)	271 (34.2)	347 (43.6)	413 (52.1)	<0.001
**Lipids §**					
Total cholesterol, mg/dl†	201 (41)	194 (35)	188 (37)	183 (37)	<0.001
LDL cholesterol, mg/dl†	127 (39)	122 (31)	117 (31)	105 (29)	<0.001
HDL cholesterol, mg/dl†	45 (12)	41 (10)	37 (10)	34 (8)	<0.001
Triglycerides, mg/dl ‡	109 (40)	135 (44)	161 (48)	224 (69)	<0.001§
LDL triglycerides, mg/dl‡	27 (10)	30 (10)	33(11)	35 (13)	<0.001§
Total apolipoprotein B, mg/dl	97 (24)	103 (22)	106 (24)	109 (26)	<0.001
LDL apolipoprotein B, mg/dl	83 (24)	86 (21)	87 (22)	85 (22)	0.002
LDL apolipoprotein B to LDL cholesterol ratio	0.66 (0.05)	0.71 (0.07)	0.75 (0.04)	0.81 (0.06)	-
LDL diameter, nm	16.9 (0.4)	16.6 (0.4)	16.5 (0.3)	16.3 (0.4)	<0.001
**C-reactive protein**, mg/l	6.9 (18.0)	7.5 (13.9)	11.3 (22.0)	10.9 (18.9)	<0.001
**Coronary artery disease** ‖					<0.001
No	290 (37.2)	170 (21.8)	127 (16.7)	127 (16.8)	
Stable	343 (44.0)	391 (50.2)	405 (53.1)	330 (43.5)	
Acute coronary syndrome	146 (18.7)	218 (28.0)	230 (30.2)	301 (39.7)	
**NYHA functional class**					0.011
I	420 (53.0)	417 (52.6)	426 (53.6)	394 (49.7)	
II	248 (31.3)	225 (28.4)	235 (29.6)	217 (27.4)	
III	110 (13.9)	120 (15.1)	115 (14.5)	153 (19.3)	
IV	14 (1.8)	31 (3.9)	19 (2.4)	29 (3.7)	
**Left ventricular function #**					<0.001
Normal	550 (76.9)	482 (67.7)	436 (62.8)	440 (62.4)	
Midly impaired	89 (12.4)	113 (15.9)	122 (17.6)	118 (16.7)	
Moderately impaired	51 (7.1)	84 (11.8)	102 (14.7)	100 (14.2)	
Severely impaired	25 (3.5)	33 (4.6)	34 (4.9)	47 (6.7)	
**Friesinger score**					<0.001
1st quartile	277 (35.0)	167 (21.1)	129 (16.2)	120 (15.1)	
2nd quartile	205 (25.9)	172 (21.7)	165 (20.8)	177 (22.3)	
3rd quartile	193 (24.4)	262 (33.0)	289 (36.4)	285 (35.9)	
4th quartile	117 (14.8)	192 (24.2)	212 (26.7)	211 (26.6)	
**Peripheral vascular disease**	41 (5.2)	72 (9.1)	78 (9.8)	97 (12.2)	<0.001
**Cerebrovascular disease**	65 (8.2)	63 (7.9)	87 (10.9)	75 (9.5)	0.144
**Statin**	222 (28.0)	331 (41.7)	447 (56.2)	482 (60.8)	<0.001
**Non-statin lipid lowering drug**	24 (3.0)	13 (1.6)	15 (1.9)	18 (2.3)	0.255

Legend: Values are means ± standard deviations or medians (25th-75th percentiles) in cases of continuous variables and numbers (percentages) in cases of categorical data; * for differences across the 4 groups calculated with χ^2 test and ANalysis of VAriance for categorical and continuous data, respectively; † to convert to millimoles per liter, multiply by 0.02586; ‡ to convert to millimoles per liter, multiply by 0.01129; § ANalysis Of VAriance of logarithmically transformed values; ‖ 779/779/762/758; #715/712/694/705

Supplementary Table S4

Title: Baseline characteristics according to LDLapoB/$LDLC_{calc}$ quartiles in statin-naïve LURIC patients

	**1st quartile**	**2nd quartile**	**3rd quartile**	**4th quartile**	***p******
**Number**	570	462	348	311	-
**Male sex**	324 (56.8)	324 (70.1)	250 (71.8)	228 (73.3)	<0.001
**Age**, years	62.7 (11.4)	62.6 (11.1)	62.5 (11.2)	62.8 (10.5)	0.974
**Body mass index**, kg/m^2	26.4 (3.8)	27.3 (4.1)	27.8 (4.2)	28.8 (4.4)	<0.001
**Hypertension**	391 (68.6)	336 (72.7)	255 (73.3)	247 (79.4)	0.007
**Smoking**					0.001
Never	284 (49.8)	183 (39.6)	119 (34.2)	102 (32.8)	
Former smoker	201 (35.3)	196 (42.4)	157 (45.1)	144 (46.3)	
Current smoker	85 (14.9)	83 (18.0)	72 (20.7)	65 (20.9)	
**Diabetes mellitus**	146 (25.6)	161 (34.8)	162 (46.6)	165 (53.1)	<0.001
**Lipids**					
Total cholesterol, mg/dl†	204 (38)	201 (33)	198 (35)	193 (36)	<0.001
LDL cholesterol, mg/dl†	130 (36)	128 (28)	124 (27)	114 (29)	<0.001
HDL cholesterol, mg/dl†	45 (12)	41 (11)	38 (10)	34 (9)	<0.001
Triglycerides, mg/dl ‡	109 (40)	138 (45)	172 (51)	234 (69)	<0.001§
LDL triglycerides, mg/dl‡	27 (9)	30 (9)	34 (11)	37 (14)	<0.001§
Total apolipoprotein B, mg/dl	99 (23)	108 (20)	112 (22)	116 (24)	<0.001
LDL apolipoprotein B, mg/dl	85 (23)	90 (19)	92 (20)	90 (21)	<0.001
LDLapolipoprotein B to LDLcholesterol ratio	0.66 (0.05)	0.71 (0.09)	0.74 (0.04)	0.80 (0.06)	-
LDL diameter, nm	16.9 (0.4)	16.6 (0.4)	16.5 (0.4)	16.3 (0.4)	<0.001
**C-reactive protein**, mg/l	5.7 (13.9)	7.6 (14.1)	9.3 (17.0)	10.3 (17.2)	<0.001§
**Coronary artery disease** ||					<0.001
No	255 (45.3)	149 (32.6)	103 (30.3)	91 (29.8)	
Stable Angina	234 (41.6)	212 (46.4)	164 (48.2)	135 (44.3)	
ACS	74 (13.1)	96 (21.0)	73 (21.5)	79 (25.9)	
**NYHA functional class**					0.018
I	300 (52.6)	232 (50.2)	176 (50.)	138 (44.4)	
II	184 (32.3)	132 (28.6)	113 (32.5)	97 (31.2)	
III	77 (13.5)	78 (16.9)	50 (14.4)	60 (19.3)	
IV	9 (1.6)	20 (4.3)	9 (2.6)	16 (5.1)	
**Left ventricular function #**					0.884
Normal	414 (79.3)	288 (69.4)	202 (65.4)	176 (64.0)	
Midly impaired	58 (11.1)	60 (14.5)	48 (15.5)	40 (14.5)	
Moderately impaired	34 (6.5)	45 (10.8)	41(13.3)	36 (13.1)	
Severely impaired	16 (3.1)	22 (5.3)	18 (5.8)	23 (8.4)	
**Friesinger score**					<0.001
1st quartile	239 (41.9)	144 (31.2)	100 (28.7)	90 (28.9)	
2nd quartile	167 (29.3)	116 (25.1)	95 (27.3)	87 (28.0)	
3rd quartile	109 (19.1)	123 (26.6)	90 (25.9)	77 (24.8)	
4th quartile	55 (9.6)	79 (17.1)	63 (18.1)	57 (18.3)	
**Peripheral vascular disease**	26 (4.6)	34 (7.4)	27 (7.8)	34 (10.9)	0.005
**Cerebrovascular disease**	45 (7.9)	35 (7.6)	43 (12.4)	27 (8.7)	0.076
**Non-statin lipid lowering drugs**	18 (3.2)	12 (2.6)	10 (2.9)	9 (2.9)	0.963

Legend: Values are means ± standard deviations or medians (25th-75th percentiles) in cases of continuous variables and numbers (percentages) in cases of categorical data; * for differences across the 4 groups calculated with χ^2 test and ANalysis of VAriance for categorical and continuous data, respectively; † to convert to millimoles per liter, multiply by 0.02586; ‡ to convert to millimoles per liter, multiply by 0.01129; § ANalysis Of VAriance of logarithmically transformed values; || 563/457/340/305; # 522/415/309/275

Supplementary Table S5

Title: Baseline characteristics according to LDLapoB/$LDLC_{calc}$ quartiles in LURIC patients on statins

	1st quartile	2nd quartile	3rd quartile	4th quartile	*p**
**Number**	222	331	447	482	-
**Male sex**	91 (41.0)	82 (24.8)	111 (24.8)	119 (24.7)	<0.001
**Age**, years	64.7 (10.0)	63.5 (10.1)	62.9 (9.6)	61.9 (10.1)	0.004
**Body mass index**, kg/m^2	26.4 (3.7)	26.8 (3.6)	27.7 (3.9)	28.4 (4.2)	<0.001
**Hypertension**	147 (66.2)	219 (66.2)	329 (73.6)	369 (76.6)	0.002
**Smoking**					0.015
Never	89 (40.1)	110 (33.2)	143 (32.0)	126 (26.1)	
Former smoker	94 (42.3)	156 (47.1)	212 (47.4)	238 (49.4)	
Current smoker	39 (17.6)	65 (19.6)	92 (20.6)	118 (24.5)	
**Diabetes mellitus**	63 (28.4)	110 (33.2)	185 (41.4)	248 (51.5)	<0.001
**Lipids**					
Total cholesterol, mg/dl†	192 (45)	184 (37)	181 (38)	176 (37)	<0.001
LDL cholesterol, mg/dl†	120 (44)	114 (34)	112 (32)	100 (28)	<0.001
HDL cholesterol, mg/dl†	43 (11)	41 (10)	37 (9)	34 (8)	<0.001
Triglycerides, mg/dl ‡	110 (41)	130 (43)	152 (45)	217 (69)	<0.001§
LDL triglycerides, mg/dl ‡	28 (10)	29 (10)	32 (11)	34 (13)	<0.001§
Total apolipoprotein B, mg/dl	93 (26)	97 (23)	102 (25)	105 (26)	<0.001
LDL apolipoprotein B, mg/dl	79 (27)	81 (23)	84 (23)	81 (22)	0.085
LDLapolipoprotein B to LDLcholesterol ratio	0.67 (0.04)	0.72 (0.04)	0.76 (0.04)	0.82 (0.06)	-
LDL diameter, nm	16.9 (0.4)	16.6 (0.4)	16.5 (0.3)	16.3 (0.4)	<0.001
**C-reactive protein**, mg/l	10.1 (25.5)	7.4 (13.7)	12.8 (25.1)	11.3 (19.9)	<0.001§
**Coronary artery disease** ||					<0.001
No	35 (16.2)	21 (6.5)	24 (5.7)	36 (7.9)	
Stable Angina	109 (50.5)	179 (55.6)	241 (57.1)	195 (43.0)	
Acute coronary syndrome	72 (33.3)	122 (37.9)	157 (37.2)	222 (49.0)	
**NYHA functional class**					0.435
I	120 (54.1)	195 (55.9)	250 (55.9)	256 (53.1)	
II	64 (28.8)	93 (28.1)	122 (27.3)	120 (24.9)	
III	33 (14.9)	42 (12.7)	65 (14.5)	93 (19.3)	
IV	5 (2.3)	11 (3.3)	10 (2.2)	13 (2.7)	
**Left ventricular function #**					0.369
Normal	136 (70.5)	194 (65.3)	234 (60.8)	264 (61.4)	
Midly impaired	31 (16.1)	53 (17.8)	74 (19.2)	78 (18.1)	
Moderately impaired	17 (8.8)	39 (13.1)	61 (15.8)	64 (14.9)	
Severely impaired	9 (4.7)	11 (3.7)	16 (4.2)	24 (5.6)	
**Friesinger score**					<0.001
1st quartile	38 (17.1)	23 (6.9)	29 (6.5)	30 (6.2)	
2nd quartile	38 (17.1)	56 (16.9)	70 (15.7)	90 (18.7)	
3rd quartile	84 (37.8)	139 (42.0)	199 (44.5)	208 (43.2)	
4th quartile	62 (27.9)	113 (34.1)	149 (33.3)	154 (32.0)	
**Peripheral vascular disease**	15 (6.8)	38 (11.5)	51 (11.4)	63 (13.1)	0.106
**Cerebrovascular disease**	20 (9.0)	28 (8.5)	44 (9.8)	48 (10.0)	0.883
**Non-statin lipid lowering drugs**	6 (2.7)	1 (0.3)	5 (1.1)	9 (1.9)	0.087

Legend: Values are means ± standard deviations or medians (25th-75th percentiles) in cases of continuous variables and numbers (percentages) in cases of categorical data; * for differences across the 4 groups calculated with χ^2 test and ANalysis of VAriance for categorical and continuous data, respectively; † to convert to millimoles per liter, multiply by 0.02586; ‡ to convert to millimoles per liter, multiply by 0.01129; § ANalysis Of VAriance of logarithmically transformed values; || 216/322/422/453; # 193/297/385/430

Supplementary Table S6

Title: Baseline characteristics according to LDLapoB/$LDLC_{calc}$ quartiles in the replication cohort

	1st quartile	2nd quartile	3rd quartile	4th quartile	*p* *
**Number**	415	415	415	415	
**Male sex**	227 (54.7)	284 (68.4)	276 (66.5)	305 (73.5)	<0.001
**Age**	66.2 (10.7)	65.3 (10.5)	63.9 (10.1)	62.3 (10.4)	<0.001
**Body mass index**, kg/m^2	27.4 (4.5)	27.8 (4.4)	27.2 (4.3)	27.6 (4.2)	0.463
**Hypertension**	287 (69.2)	291 (70.1)	250 (60.2)	229 (55.2)	<0.001
**Smoking**	223 (53.7)	248 (59.8)	239 (57.6)	266 (64.1)	0.008
**Diabetes mellitus**	81 (19.5)	118 (28.4)	124 (29.9)	154 (37.1)	<0.001
**Lipids**					
Total cholesterol, mg/dl†	204 (47)	196 (45)	205 (47)	207 (42)	0.151
LDL cholesterol, mg/dl†	135 (43)	128 (40)	130 (38)	122 (34)	<0.001
HDL cholesterol, mg/dl†	62 (16)	56 (15)	51 (14)	45 (14)	<0.001
Triglycerides, mg/dl ‡	115 (49)	131 (68)	135 (66)	172 (80)	<0.001
Apolipoprotein B,mg/dl	78 (21)	87 (23)	102 (26)	114 (26)	<0.001
LDLapoB, mg/dl	62 (21)	73 (22)	88 (26)	99 (26)	<0.001
LDLapoB/$LDLC_{calc}$	0.48 (0.04)	0.57 (0.02)	0.67 (0.03)	0.82 (0.09)	-
**C-reactive protein**, mg/l	3 (6)	4 (7)	5 (6)	10 (17)	<0.001
**Coronary artery disease**					
Any	321 (77)	341 (82.2)	326 (78.6)	355 (85.5)	0.008
Significant	204 (49.2)	240 (57.8)	238 (57.3)	269 (64.8)	<0.001
**Statin**	171 (41.2)	198 (47.7)	200 (48.2)	191 (46)	0.109

Legend: Values are means (standard deviation) for continues variables and numbers (percentages) for categorical variables. * for differences across the 4 groups calculated with χ^2 test for categorical variables and Jonckheere Terpstra Test for continuous variables. † to convert to millimoles per liter, multiply by 0.02586; ‡ to convert to millimoles per liter, multiply by 0.01129

Supplementary Table S7

Title: Cardiovascular mortality according to LDLapoB/$LDLC_{calc}$ quartiles in the entire replication cohort

			Model 1 *		Model 2†	
	**N**	**CD (%)**	**HR (95% CI)**	**P**	**HR (95% CI)**	**P**
1st quartile	409	30 (7.3)	1.0 reference	-	1.0 reference	-
2nd quartile	407	52 (12.8)	1.48 (0.79-2.77)	0.216	1.41 (0.76-2.64)	0.279
3rd quartile	411	60 (14.6)	2.00 (1.10-3.61)	0.021	1.64 (0.90-3.02)	0.131
4th quartile	413	79 (19.1)	2.70 (1.55-4.69)	<0.001	1.97 (1.09-3.55)	0.024

Legend: N number, CD cardiovascular death; HR hazard ratio (calculated with Cox regression); CI confidence interval; * adjusted for sex, age, statin use, and the interaction between statin use and LDLapoB/$LDLC_{calc}$ quartiles; † model 1 with additional adjustment for body mass index, hypertension, diabetes, HDL cholesterol, triglycerides, and smoking.
